# Ultrasound-guided bursal injections

**DOI:** 10.1007/s00256-022-04153-y

**Published:** 2022-08-26

**Authors:** Kevin C. McGill, Rina Patel, David Chen, Nikki Okwelogu

**Affiliations:** 1 Department of Radiology and Biomedical Imaging, University of California, San Francisco, CA USA; 2 Department of Radiology, University of California, Davis, CA USA; 3 University of California, San Francisco, CA USA

**Keywords:** Bursa, Ultrasound, Injection, Aspiration, Steroid

## Abstract

The native bursa is a structure lined by synovium located adjacent to a joint which may serve to decrease friction between the tendons and overlying bone or skin. This extra-articular structure can become inflamed resulting in bursitis. Steroid injections have proven to be an effective method of treating bursal pathology in various anatomic locations. Performing these procedures requires a thorough understanding of relevant anatomy, proper technique, and expected outcomes. Ultrasound is a useful tool for pre procedure diagnostic evaluation and optimizing needle position during these procedures while avoiding adjacent structures. The purpose of this article is to review core principles of ultrasound-guided musculoskeletal procedures involving bursae throughout the upper and lower extremities.

## Introduction

Utilization of diagnostic musculoskeletal ultrasound and ultrasound-guided procedures has steadily increased over the past few years, most significantly among non-radiologist providers [1–3]. Ultrasound provides an opportunity for practitioners to both diagnose and treat a large variety of musculoskeletal conditions in one interaction and while proving to be a useful tool for essentially all bursal injections or aspirations of the extremities.

The native bursa is a structure lined by synovium located adjacent to a joint which may serve to decrease friction between the tendons and overlying bone or skin. Alternatively, an adventitial bursa is a non-native structure which develops later in life secondary to abnormal stress and friction involving subcutaneous soft tissue. Inflammation of these extra-articular structures results in bursitis which are usually treated conservatively with non-invasive treatments such as anti-inflammatory medications or physical therapy. If these methods are unsuccessful, more invasive options such as percutaneous needle-based or surgical interventions are considered.

The purpose of this article is to review core principles of musculoskeletal interventions involving selected bursae using ultrasound guidance.

## Peri-procedural recommendations

### Pre-procedural evaluation and consent

Following a review of the relevant imaging and medical history, including lab values, allergies, and current medications, informed consent is required prior to any procedure. At the authors’ institution, bursal injections are considered to demonstrate a low-risk for bleeding, therefore the Society of Interventional Radiology recommendations [4] regarding lab values for low-risk procedures are followed. As per the guidelines, screening anticoagulation laboratory values are not recommended for patients with minimal risk factors. For patients with increased risk, the international normalized ratio should be corrected within the range of 2.0–3.0 or less with a platelet count of < 20 × 10^9^/L. Immediately prior to the procedure, a short pause, also known as a “time out”, is always performed to confirm the correct patient, procedure, and site in addition to reconfirmation of relevant allergies, medications, and any abnormal lab values.

Contraindications for ultrasound-guided soft tissue injections may include prior allergic reaction and cellulitis or soft tissue infection at the injection site. Other, more easily modifiable contraindications, such as timing of surgery or immunizations should be discussed with the patient and referring physician prior to the procedure.

In most musculoskeletal interventions, the only medications administered are a local anesthetic and a corticosteroid [5] (Table 1). Side effects are usually mild and may include facial flushing, skin reaction/hypopigmentation, soft tissue atrophy, steroid flare, transient increase in blood sugar [6, 7], adverse psychiatric effects [8, 9], and allergic reaction ranging from mild to anaphylaxis. Allergy to injectable steroids is rare and may be related to a medication additive rather than the steroid itself [10]. For extra-articular steroid injections, major adverse events have been reported as occurring 0–5.8% with minor adverse events ranging from 0 to 81% [11]. Pain following injection is the most common minor adverse event [11], described as a transient issue, not requiring intervention. Infection is an extremely rare complication, with an incidence rate of 0.0046% [12].

**Table 1:** Commonly used injectable steroids and local anesthetics

Steroids	Anesthetics
Methylprednisolone acetate (40 mg/mL or 80 mg/mL)	Lidocaine (1%)
Triamcinolone acetonide (40 mg/mL)	Bupivacaine (0.25%)
Betamethasone acetate (6 mg/mL)	Ropivacaine (0.5%)
Dexamethasone sodium phosphate (4 mg/mL)	

### Medication choice

While injectable steroids are frequently used to treat pain, there is no consensus regarding the superiority of a specific agent, for bursal injections. Commonly used steroids, however, do contain slightly different properties. For example, particulate steroids such as methylprednisone, triamcinolone and betamethasone have lower solubility and therefore, theoretically last longer at the injection site compared to non-particulates such as dexamethasone.

While allergic reaction is always a concern, the evidence of cross reactivity within the amide group is inconsistent [13, 14]. In the case of a documented allergy to lidocaine or other amide group anesthetic, such as bupivacaine or ropivacaine, an anesthetic from the ester group, such as chloroprocaine, can be considered. Due to the ubiquity of lidocaine in medical procedures, allergen testing should also be considered for severe reactions.

### Technique

Focused pre-procedural scanning, including grayscale and Doppler imaging, should be performed before every procedure. This provides the opportunity to choose the optimal approach and assess for any additional clinically relevant pathology. For example, the presence of a rotator cuff tear identified during pre-procedural scanning for a subacromial subdeltoid (SASD) bursa injection may necessitate discussion with the referring provider due to the association between steroid injections prior to rotator cuff repair and increased rates of infection or revision surgery [15–18].

The majority of bursal injections can be performed with a 9–15 MHz linear transducer. A curved low-frequency (< 7 MHz) transducer may be useful for larger patients and deeper targets, while a high-frequency small footprint linear array transducer (> 15 MHz) can be used for smaller, superficial targets, especially involving the digits. Optimization of the ultrasound settings including changing patient position or using specific advanced applications on newer ultrasound machines can improve visualization of the target.

For most procedures, either a 22-gauge or 25-gauge needle is sufficient, although for aspiration of potentially viscous fluid, a larger gauge needle (16–18 gauge) is recommended. For most ultrasound injections, a test injection of lidocaine may be useful to confirm precise placement of the needle prior to steroid injection. This is especially beneficial for injecting bursae, which can be difficult to identify when not distended with fluid.

While image-guided bursal injections can be challenging, especially when bursal fluid is absent, they have been shown to be more accurate than injections by palpation alone at certain anatomic sites [19]. If the bursa is thickened or distended, it can easily be targeted for aspiration and/or injection. When absent, anatomic landmarks and dynamic technique [20] may improve localization. Initial slow injection of a very small amount of the anesthetic or steroid/anesthetic mixture may distend the bursa and provide feedback for possible needle re-localization prior to injecting the full dose. When injecting the bursa, there should be a loss of resistance with fluid flowing away from the needle. The injectate including anesthetic and steroid should be slightly more echogenic than the anesthetic alone.

### Post-procedural guidance

At the author’s institution, following the injection, a small bandage is placed over the injection site and the patient is instructed to keep the area dry for at least 24 h. An immediate post-procedure pain assessment is provided by the patient using a 0- to10-point scale with comparison to pre procedure levels. The patient may also be asked to perform any provocative maneuvers or positions which exacerbate the pain to provide a functional assessment. Patients are informed that it may take up to 7 days for the steroids to reach their full effect and they are asked to continue to monitor their pain closely over the next few weeks to determine the efficacy of the injection.

## Subacromial/subdeltoid bursa

### Background

The SASD bursa, the largest bursa in the body, is composed of 2 separate bursae, the subacromial and subdeltoid bursae, which are contiguous in 95% of patients [21]. The SASD bursa is a large but thin structure, even in patients with bursitis where the average thickness is no more than 2 mm [22]. A 2007 study by Tsai reviewed shoulder ultrasounds of 268 patients with unilateral shoulder pain and either Neer stage 1 or stage 2 subacromial impingement. Comparing the symptomatic and asymptomatic sides, a statistically significant difference in thickness of the SASD bursa was identified (mean thickness 1.27 mm versus 0.75 mm) [23].

SASD bursitis is often related to subacromial impingement but can also have other causes such as inflammatory arthritis, crystal deposition, or infection. Impingement can be related to overhead activities, degenerative changes within the acromioclavicular joint, hypertrophy of the coracoacromial ligament, and anatomic variations of the acromion including differences in morphology and the presence of an os acromiale.

Ultrasound guidance allows the radiologist to perform both a diagnostic exam of the shoulder and a dynamic evaluation of subacromial impingement. In patients with ultrasound evidence of SASD bursitis, hypoechoic intrabursal fluid may be present in between hyperechoic peribursal fat. Bunching of the bursa during shoulder abduction may demonstrates evidence of subacromial impingement.

Although a subacromial bursal injection is routinely performed under palpation and ultrasound guidance, ultrasound is recommended to maximize accuracy [24]. A systematic review and meta-analysis of randomized controlled trials concluded steroid injection to be superior to physiotherapy for decreasing pain and improving shoulder function 6–7 weeks following the procedure [25]. This procedure is expected to reduce pain, enabling patients to continue physical therapy and participate in their activities of daily living [26] which makes steroid injections an effective short-term treatment for subacromial impingement.

### Technique

**Fig. 1:**
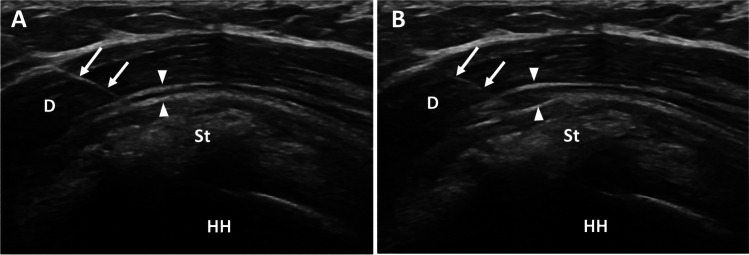
Subacromial bursa injection. Findings: long axis view of the supraspinatus tendon (St) before (**A**) and after (**B**) injection of local anesthetic into the subacromial/subdeltoid bursa outlined by thin echogenic peribursal fat (arrowheads). The needle (arrows) traverses the deltoid (**D**) muscle with tip located within the bursa superficial to the supraspinatus tendon (St) and humeral head (HH). The supraspinatus tendon contains echogenic calcifications, consistent with calcium hydroxyapatite deposition

The SASD bursa can be accessed through a lateral deltoid approach, far away from any large neurovascular structures. The patient may be sitting upright or supine with their arm adducted and in neutral or internally rotated. The needle is slowly advanced lateral to medial until the tip of the needle is at the peri-bursal fat. A small amount of anesthetic is injected which should expand the bursa, separating the peri-bursal fat planes (Fig. 1) and may also be seen extending laterally superficial to the supraspinatus insertion on the greater tuberosity. Given the large potential space of the bursa, the fluid may quickly disperse away from the needle delaying bursal distention. If the fluid continues to collect around the needle without expanding the rest of the bursa, it is possible that the needle tip is in the deltoid or supraspinatus and therefore should be repositioned. Once the location is confirmed a mixture of 40 mg of triamcinolone or methylprednisolone and 2 mL of bupivacaine or ropivacaine should be injected.

## Scapulothoracic bursa

### Background

The scapulothoracic articulation is a complex pseudojoint created by the large flat scapula sliding over the thorax without bony attachments. There are two anatomic (major) bursae including the infraserratus (scapulothoracic) and supraserratus (subscapularis) bursae in addition to four inconsistent adventitial (minor) bursae [27]. The infraserratus bursa, located between the serratus anterior and chest wall, is the most common source for scapulothoracic bursitis [28] and therefore the primary target for steroid injections.

Scapulothoracic pain is an uncommon but potentially debilitating cause of shoulder pain. There are a number of different etiologies, including repetitive overhead activity, snapping scapula syndrome, scapular dyskinesia, or altered biomechanics [29]. In a study by Conduah et al., 43% of cases of scapulothoracic bursitis were associated with an anatomic abnormality [30]. Nonoperative management is generally recommended prior to surgical intervention which may include scapulothoracic bursectomy or medial angle resection [31].

Injection of the scapulothoracic bursa (STB) under ultrasound guidance allows dynamic visualization of the needle and lung/pleura during the procedure. Ultrasound also may identify other related soft tissue pathology such as fibrous adhesions, scarring, or even bursal fluid, which should be aspirated prior to an injection [32].

In addition to performing a diagnostic evaluation with ultrasound, prior imaging should be reviewed to assess for other potential causes of bursitis or scapulothoracic snapping such as elastofibroma dorsi [33–35], osteochondroma, prior scapula/rib fracture, or scoliosis [27, 36]. Other mimickers for scapulothoracic bursitis include referred pain from the shoulder or lower cervical spine.

A retrospective study by Adler [32] of 22 patients receiving STB injections showed improvement in 82% of patients following a steroid injection. Promising results were also obtained by Holder et al. [37] and Chang et al. [38] with the latter demonstrating consistent improvement in VAS scores taken 1, 2, and 3 weeks after treatment. Steroid injections have proven to be effective in treating scapulothoracic pain and should be considered in patients with scapulothoracic bursitis and snapping scapula.

### Technique

**Fig. 2:**
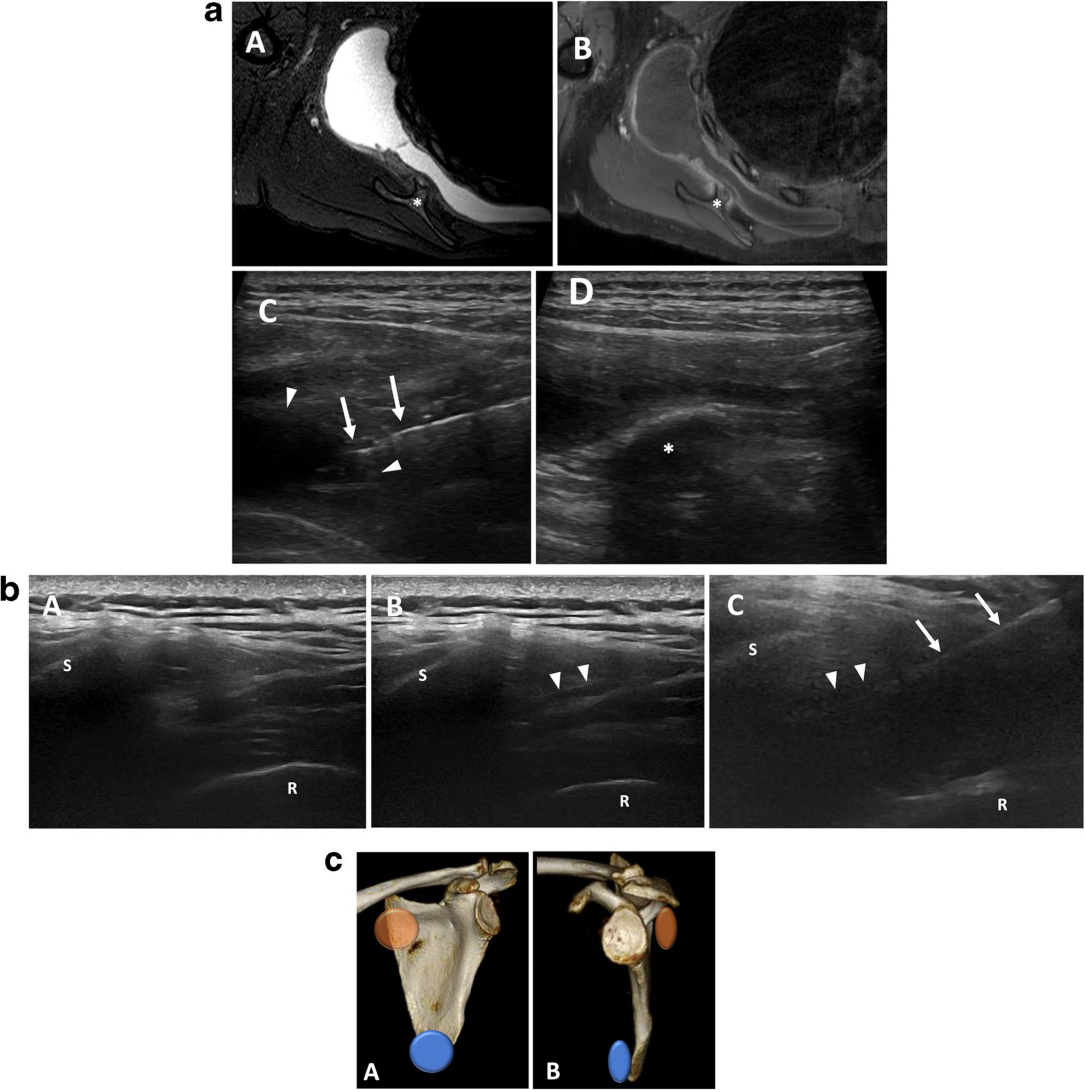
**a **Scapulothoracic bursitis. Findings: axial T2 fat-saturated (A) and T1 fat-saturated post-contrast (B) MRI of the shoulder with large scapulothoracic bursal effusion secondary to osteochondroma (asterisk). Ultrasound prior to aspiration (C) demonstrates needle (arrows) within the heterogenous fluid collection within the bursa (arrowheads). Post-aspiration ultrasound image (D) shows near complete resolution of fluid adjacent to the osteochondroma (asterisk). **b** Scapulothoracic bursal fluid with dynamic maneuver. Findings: with the transducer in transverse over the medial scapula, the scapula margin (S) and adjacent rib (R) are visualized. A patient with scapulothoracic pain demonstrates no sonographic abnormality with arm at side (A). When the arm is flexed behind the back (B), a small amount of fluid is visualized in the scapulothoracic bursa (arrowheads) confirming the diagnosis of scapulothoracic bursitis. The arm remains flexed during steroid injection (C) with needle (arrow) trajectory nearly parallel to scapula. **c** Major scapulothoracic bursae. Findings: three-dimensional frontal and lateral images of the scapula demonstrating the anatomic locations of the major scapulothoracic bursae. Inferaserratus/scapulothoracic bursa (blue), supraserratus/subscapularis bursa (orange)

Injection is typically performed while prone with the symptomatic upper extremity in the chicken wing position. The arm is placed across the lower back, adducted with the elbow flexed, widening the scapulothoracic interval. The ultrasound probe is then placed over the area of pain, typically along the medial inferior border of the scapula. The normal scapulothoracic bursa is very thin and difficult to visualize. Even when distended with a thin layer of hypoechoic fluid, the majority of the scapulothoracic bursa is obscured by the bony scapula and challenging to access with ultrasound. It is important to maintain a needle trajectory which is parallel with the scapula and/or near parallel to the curved chest wall. A 25-gauge needle or 22-gauge spinal needle can be advanced into the bursa while slowly administering a mixture of 40 mg of triamcinolone or methylprednisolone and 4 mL of ropivacaine until the bursa distends (Fig. 2). If the patient develops respiratory symptoms during or immediately following the injection, a follow-up chest radiograph should be obtained to assess for pneumothorax.

## Iliopsoas bursa

### Background

The iliopsoas muscle is a compound muscle composed of the psoas major, psoas minor, and the iliacus. The iliacus and psoas originate from the iliac fossa and vertebral bodies respectively, extending inferiorly toward the insertion of the psoas on the lesser trochanter of the femur where it serves as the main flexor of the hip. The iliopsoas bursa, the largest bursa around the hip, is located deep to the myotendinous junction of the iliopsoas muscle, anterior to the hip capsule, and lateral the femoral vessels. Pain related to the psoas tendon/iliopsoas bursa may be present secondary to tendinosis, which is commonly seen in the setting of overuse, acute trauma, or following total hip arthroplasty [39, 40]. Snapping hip syndrome, another cause of hip pain related to psoas tendon, is a condition where there is pain and an audible or perceived snapping of the hip with movement. While abnormal motion of the psoas tendon can be identified in the patients on dynamic evaluation, static sonography in symptomatic hips is typically normal [40]. Conversely, iliopsoas impingement syndrome in patients with total hip arthroplasty is associated with certain ultrasound findings including visibility of the anterior cup, contact between the anterior cup and psoas tendon, iliopsoas tendinopathy, and iliopsoas bursitis [41].

While peritendinous steroid injection have proven to be effective when performed with ultrasound or fluoroscopy guidance [39, 40, 42], sonographic guidance decreases the risk of injury soft tissue structures including the nearby neurovascular bundle. Steroid injections have also proven effective in treating this pain regardless of the presence of additional intraarticular pathology [32, 42, 43]. Studies have also demonstrated decreased pain after steroid injections in patients with and without hip arthroplasty [39, 44]. A large prospective study of ultrasound injections in 178 patients also demonstrated positive results with statistically significant improvement in both sports and recreation and quality of life (QOL) scores measured prior to and 6 weeks following the procedure [42].

### Technique

**Fig. 3:**
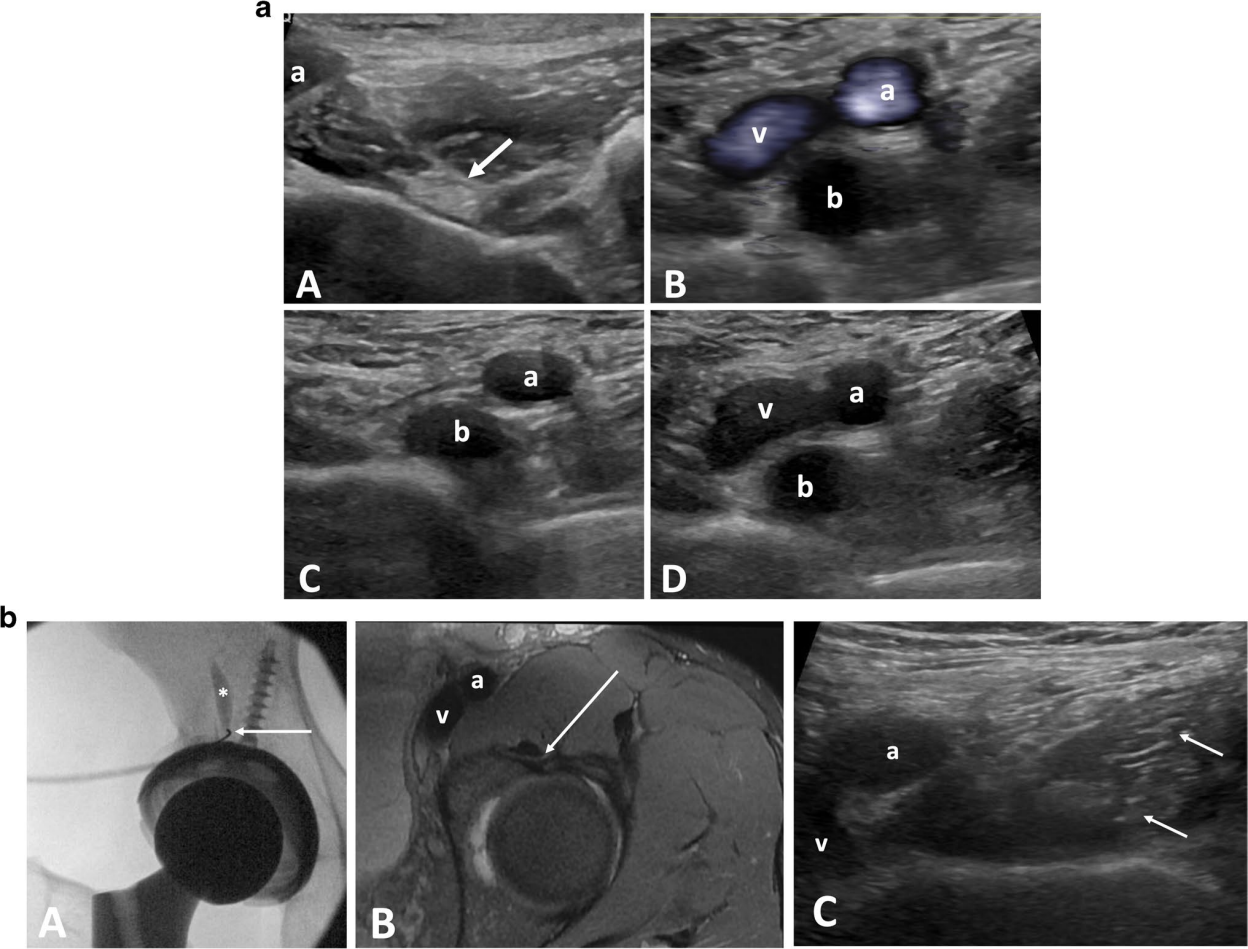
**a** Psoas bursal effusion. Findings: transverse grayscale ultrasound of the hip (A) demonstrates a normal psoas tendon (arrow) and femoral artery (a). Transverse views (B), (C), and (D) of the hip in a patient with a bursal effusion. In (B), Power Doppler images demonstrates a bursal effusion (b) with flow in the adjacent femoral artery (a) and vein (v). (C) and (D) were obtained with and without compression. In (C) the vein is compressed while the bursal effusion is unchanged. **b** Iliopsoas tendon sheath injection. Findings: fluoroscopic images of the right hip (A) in a patient with a total hip arthroplasty during iliopsoas bursa injection. Contrast (asterisk) moves superiorly away from the needle (arrow) confirming location of the within the iliopsoas bursa. Axial T2 fat-saturated and transverse ultrasound images (B and C) of the left hip on a different patients demonstrate the needle trajectory (arrow) during an ultrasound guided injection

For the standard technique, the patient is positioned supine (Fig. 3) and the transducer is placed in transverse plane at the acetabular brim. The iliopsoas tendon should be identified as an elliptical echogenic structure medial and superficial to the joint capsule along the lateral margin of the iliopectineal eminence of the acetabulum [32]. The femoral neurovascular bundle can be identified medial to the iliopsoas tendon and thus avoided during injection. Traversing lateral to medial, a 22-gauge spinal needle is advanced into the bursa. A steep trajectory is required to guide the tip of the needle to the final location, deep to the tendon, in between the tendon and acetabular brim, prior to injection of a mixture of 40 mg of triamcinolone or methylprednisolone and 4 mL of ropivacaine. Images taken following the injection should demonstrate distention of the iliopsoas bursa and/or microbubbles in a peritendinous distribution.

## Trochanteric bursa

### Background

The trochanteric (subgluteus maximus) bursa is deep to the gluteus maximus and tensor fascia lata at the level of the greater trochanter. It is the largest lateral hip bursa, overlying the attachment of the gluteus medius, gluteus minimus, and vastus lateralis [45]. There are multiple additional bursae about the greater trochanter, which may be variable in number, location, and histology; they are generally located deep to their respective tendons and named accordingly, including the subgluteus minimus and subgluteus medius bursae [46]. The anatomy of the bursae at the greater trochanter is complex, including both native bursae and non-native bursae which can be acquired due to increased hip offset [47] or excessive friction [45].

Greater trochanteric pain syndrome is a common cause of lateral hip pain with an annual incidence of up to 1.8 or 1000 [48], most commonly occurring in patients 40–60 years old with a female predominance [49–52]. Trochanteric bursal injections are considered in patients with lateral hip pain, whether related to gluteus medius/minimus tendinopathy, trochanteric bursitis, or as part of a barbotage procedure to treat calcific tendinosis [20].

Both image- and landmark-guided techniques have been shown to be effective with pain reduction at 3–6 months [53–55], but with either nonexistent [56, 57] or limited effect on long-term pain reduction [58, 59]. Many of these procedures however were performed using the more common landmark or fluoroscopy guidance which tend to result in injections into the trochanteric bursa or subgluteus medius bursa [54, 60].

McEvoy et al. [56] retrospectively reviewed 65 ultrasound guided trochanteric bursal injections identifying the exact location of the injection as either in the trochanteric bursa or the subgluteus medius bursa. While injections directly into the trochanteric bursa resulted in a statistically significant decrease in pain on a visual analog scale, injections into the subgluteus medius bursa demonstrated no difference in pain following the injection. The results of the study suggest that the use of ultrasound guidance can improve outcomes of these injections by directly targeting only the trochanteric bursa. This is a unique advantage of ultrasound guidance that may improve efficacy of steroid injections [56].

There is also evidence that other musculoskeletal pathology may contribute to the outcomes of trochanteric bursa injections. Park et al. [57] investigated this in a retrospective study of 137 patients who were assessed 1, 3, and 6 months after ultrasound guided trochanteric bursa steroid injections. This study found consistent statistically significant decreases in pain and improvement in hip function at each time interval. Poor outcomes at 6 months following the injections were correlated with knee osteoarthritis and low back pain, specifically involving the facet or sacroiliac joints.

### Technique

**Fig. 4:**
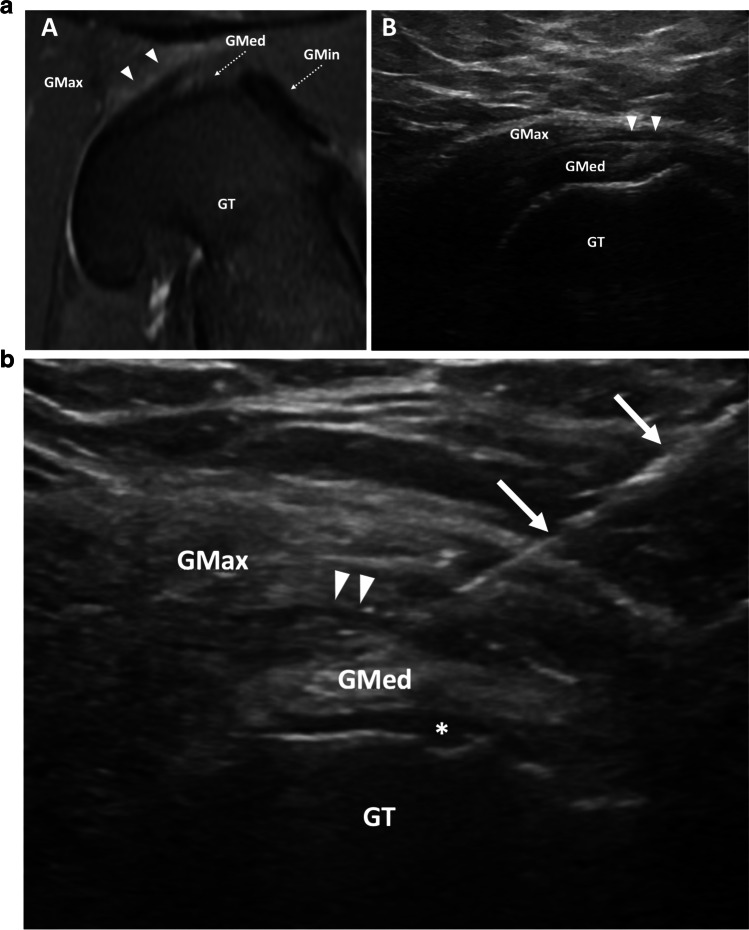
**a** Trochanteric bursitis. Findings: rotated Axial T2 fat saturated MRI of the hip (A) demonstrates edema-like signal in the greater trochanteric bursa (arrowheads) between the gluteus medius (GMed, dashed arrow) and gluteus maximus (GMax) overlying the greater trochanter (GT). Transverse sonographic view of the lateral hip (B) demonstrates trace fluid within the greater trochanteric bursa (arrowheads). Fluid is not frequently visualized in patients for whom greater trochanter bursa injection is requested. Gmin, gluteus minimus (dashed arrow). **b** Trochanteric bursa injection. Transverse ultrasound of the lateral hip demonstrates injection of fluid into the trochanteric bursa (arrowheads). Fluid in the subgluteus medius bursa (asterisk). Needle (arrows), GT, greater trochanter; GMax, gluteus maximus; GMed, gluteus medius

To perform this procedure, the patient is placed in the contralateral lateral decubitus position. The transducer is placed in the transverse plane at the greater trochanter where both the gluteus medius and minimus insertions are visualized at the lateral and anterior facets, respectively. The greater trochanter bursa is typically seen as a thin, echogenic line, deep to the gluteus maximus tendon and iliotibial band but superficial to the gluteus medius tendon attachment. A similar technique to the one initially described by Murray et al. [61] is performed in lateral decubitus position with the ipsilateral leg up and the contralateral leg bent to provide stability. The probe is placed in a transverse orientation at the level of the greater trochanter on the symptomatic hip with the foot externally rotated. The needle is advanced, posterior to anterior, through the iliotibial band, and the patient is asked to internally rotate their hip. This pulls the gluteus minimus, medius, and needle tip anteriorly guiding the needle tip into an optimal position within the trochanteric bursa. Confirmation of accurate needle placement is performed prior to injection of a mixture of 40 mg of triamcinolone or methylprednisolone and 2 mL of ropivacaine (Fig. 4). Choosing an approach which maintains a shallow angle between the probe and needle is preferred. This improves needle visualization while maximizing the distance over which the needle intersects the bursa. Since there are multiple bursae in this area which could potentially be inflamed, the recommended needle placement is wherever there is the most prominent bursal fluid. When no fluid is visualized, which is common, injection into the trochanteric bursa is preferred [56].

## Ischial bursa

### Background

The ischial bursa is located between the hamstring origin on the ischial tuberosity and the gluteus maximus, superficial to the hamstring tendons. This structure, also referred to as the ischiogluteal bursa, is an inconstant adventitial bursa which may develop in adulthood secondary due to mechanical irritation from prolonged sitting. This can present as buttock pain or even a buttock mass.

Ischial bursitis is inflammation in the bursa which can occur with repetitive motion during exercises such as cycling, running, or with prolonged sitting [62, 63]. Pain can present as mass-like sensation along the buttock, or as pain radiating down the lower leg [64]. Ischial bursitis is often diagnosed after exclusion of other causes of pain at the buttock. Prior imaging should be reviewed to identify other potential causes of pain in this region such as referred pain, calcium hydroxyapatite deposition, or other masses including myxoid tumor, schwannoma, or neurofibroma [63].

Ischial bursitis is a relatively rare condition and although there are publications describing injection technique [65, 66]; there are no studies specifically assessing clinical outcomes following ultrasound guided steroid injections into the ischial bursa. However, in a retrospective study of clinical progression and treatment of ischial bursitis by Roh et al. [67], 11 of 64 patients failed conservative therapy and underwent steroid injection. Only 2 of those patients eventually required surgery.

### Technique

**Fig. 5:**
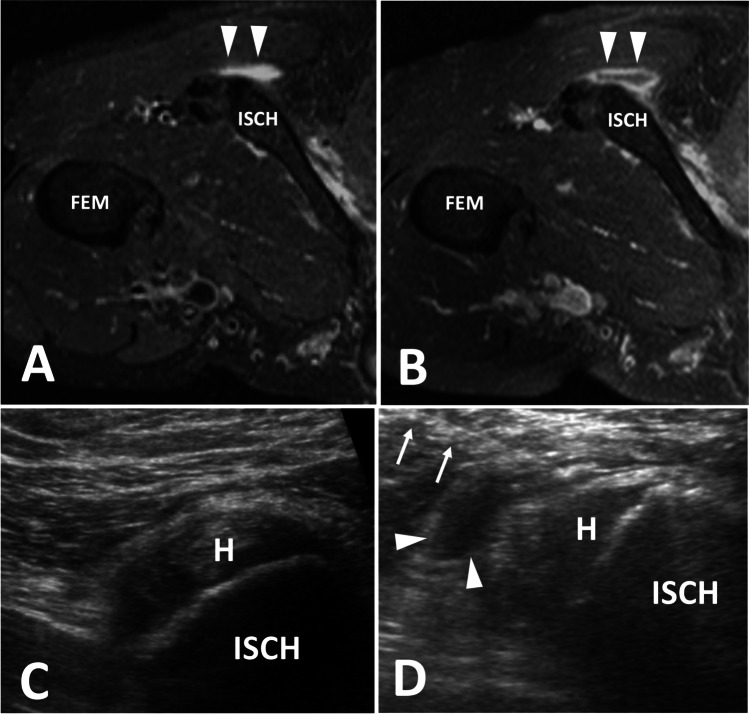
Ischial bursitis. Findings: rotated axial T2 fat-saturated (**A**) and T1 fat-saturated post contrast (**B**) images demonstrate a rim enhancing fluid collection adjacent to the ischial tuberosity within the ischial bursa, which is consistent with ischial bursitis. Transverse ultrasound images (**C**) of a different patient show the injection of echogenic steroids into the ischial bursa (arrowhead). H, hamstring origin; ISCH, ischial tuberosity; Fem, femur; needle (arrows)

Injection of the ischial bursa is typically performed with the patient in the prone position. The ultrasound probe is then used to identify the hamstring muscles which are followed superiorly to the ischial tuberosity while noting the location of the sciatic nerve (Fig. 5). The transducer is placed in the transverse plane over the most lateral aspect of the ischial tuberosity, and the needle is directed towards the space between the ischial tuberosity and gluteus maximus. Once the needle is at the peri-bursal fat, a mixture of 40 mg of triamcinolone or methylprednisolone and 4 mL of lidocaine is then injected into the bursa. Although steps are taken to avoid major neurovascular structures, it is still possible for some of the injectate to reach the sciatic nerve. Therefore, the use of shorter acting anesthetics is preferred, and patients are instructed to avoid operating a vehicle immediately following the appointment.

## Medial gastrocnemius semimembranosus bursa (Baker or popliteal cyst)

### Background

The popliteal cyst, found between the medial head of the gastrocnemius and the semimembranosus, also referred to as a Baker cyst or medial gastrocnemius semimembranosus bursa, is the most common mass of the posterior knee and a common source of knee pain. This cyst is created by a valvular communication with the posterior knee joint. Despite the common association between the presence of this structure and intra-articular pathology of up to 94% [68, 69], the treatment of the cyst with image-guided intervention often yields positive results.

Smith and colleagues reviewed the long-term outcome of 47 patients with osteoarthritis who underwent ultrasound-guided cyst aspiration, fenestration, and steroid injection and found statistically significant differences in pain scores, stiffness, and physical function without immediate or long-term complications [70]. An earlier study by Koroglu et al. [71] demonstrated similar positive results which included categorization of cysts into simple and complex. While the complex cysts were more likely to recur, all subgroups demonstrated clinical improvement with long-term follow-up averaging > 90 weeks. Post-aspiration steroid injection may be done; however, the effect is unclear since some studies suggest that the rate of recurrence may not vary between those who get corticosteroid injection after aspiration versus aspiration alone [72, 73].

### Technique

**Fig. 6:**
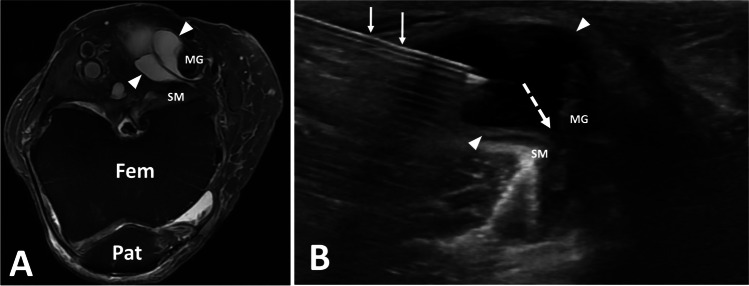
Popliteal cyst aspiration. Findings: axial T2 fat-saturated image (**A**) of the left knee with septated popliteal cyst (arrowheads). Transverse ultrasound image of the posterior knee during popliteal cyst aspiration show cyst extending toward knee joint (dashed arrow). Needle (arrow), Fem, femur; Pat, patella; SM, semimembranosus; MG, medial gastrocnemius

After targeted, diagnostic scanning to confirm the presence of a cystic structure, the patient is placed in prone position. A 16- or 18-gauge needle is advanced into the fluid collection, which is then aspirated (Fig. 6). A minimal amount of fluid can be left in the cyst to facilitate visualization during subsequent steroid and anesthetic injection. Fenestration of cyst septations is also performed, especially in cases where minimal fluid is aspirated, or when patients are presenting for repeat procedure. Following aspiration, steroid injection may be considered using a mixture of 40 mg of triamcinolone or methylprednisolone and 4 mL of ropivacaine.

## Conclusion

This review highlights a variety of bursal procedures which can be performed accurately with ultrasound guidance. The utility of ultrasound for bursal injections lies in its ability to provide precise needle localization in real-time without significant safety concerns compared to other imaging modalities. Bursae may present as very thin, curved structures; therefore, choosing the optimum approach is crucial to maximizing accuracy of needle placement and efficacy of the procedure. An awareness of the expected outcomes is important when communicating with patients and referring providers to optimize patient care.
